# Draft Genome Sequence of *Chloracidobacterium* sp. CP2_5A, a Phototrophic Member of the Phylum *Acidobacteria* Recovered from a Japanese Hot Spring

**DOI:** 10.1128/genomeA.00821-17

**Published:** 2017-10-05

**Authors:** Lewis M. Ward, Shawn E. McGlynn, Woodward W. Fischer

**Affiliations:** a Division of Geological and Planetary Sciences, California Institute of Technology, Pasadena, California, USA; b Earth-Life Science Institute, Tokyo Institute of Technology, Meguro-ko, Japan

## Abstract

The phylum *Acidobacteria* contains a single known phototrophic member, *Chloracidobacterium thermophilum*, which was recovered from a hot spring metagenome from Yellowstone National Park. Here, we expand the diversity of the genus *Chloracidobacterium* with a genome recovered from a hot spring in Japan, extending the known range of this lineage to a new continent.

## GENOME ANNOUNCEMENT

The phylum *Acidobacteria* comprises widespread but enigmatic bacteria; many subdivisions have been observed in environmental sequencing data, but only a few cultured or well-characterized representatives exist (1). The only known phototroph in this phylum,* Chloracidobacterium thermophilum*, was originally recovered from metagenomic data from a Yellowstone National Park hot spring (2) and was recently isolated (3). Here, we report the draft genome of *Chloracidobacterium* sp. strain CP2_5A, recovered from a metagenome of a Japanese hot spring.

The metagenome from which this genome was recovered was described in detail by Ward (4). In brief, CP2_5A was recovered from a cone-forming microbial mat from a moderately sulfidic, alkaline hot spring at Nakabusa Onsen in Nagano prefecture, Japan. At the time of sampling, the temperature at the collection site was 32°C with a pH measurement of 8.3.

Cells were lysed and DNA was preserved in the field using Zymo Terralyzer BashingBead Matrix and Xpedition Lysis Buffer. DNA was extracted and purified with a Zymo Soil/Fecal DNA extraction kit (Zymo Research, Irvine, CA, USA) and quantified with a Qubit version 3.0 fluorimeter (Life Technologies, Inc., Carlsbad, CA, USA). Purified DNA was submitted to SeqMatic LLC (Fremont, CA, USA) for library preparation and sequencing via Illumina HiSeq technology. Raw sequences were assembled with MegaHit version 1.02 (5) and binned using MetaWatt version 3.5.2 (6). The genome was uploaded to RAST (7) for overall characterization and was assessed for completeness and contamination using CheckM (8).

The CP2_5A genome bin is estimated to be 92% complete as determined by CheckM. The genome is 3.41 Mb, with a GC content of 64.2%, and comprises 385 contigs containing 3,023 coding sequences and 52 RNAs. The 16S gene recovered from CP2_5A is 98% identical to that of *C. thermophilum* strain E. The CP2_5A genome contains a complete suite of genes for anoxygenic phototrophy, including a type 1 reaction center (RCI) and a largely complete bacteriochlorophyll synthesis pathway (only *bchM* was not recovered in the genome bin). Consistent with the photoheterotrophic lifestyle of the isolated *C. thermophilum* strain, CP2_5A does not encode carbon fixation pathways. The CP2_5A genome encodes two* bc* complexes, an alternative complex III, a low-O_2_-affinity A-family heme-copper oxidoreductase, and a *bd*-cytochrome *c*. The RCI of CP2_5A is similar to that of the *Chloracidobacterium* isolate: it branches basal to those of phototrophic *Chlorobi* spp. but more derived than the simple RCIs found in *Heliobacteria* spp. While one of the *bc* complexes in CP2_5A clusters with that of *C. thermophilum* strain E, the other is instead closely related to that from the recently discovered phototrophic phylum *Gemmatimonadetes* (9), potentially marking horizontal gene transfer between these two clades.

The degree of genomic and metabolic similarity between *Chloracidobacterium* strains found in hot springs across at least two continents suggests a broadly conserved ecology between microbial lineages in similar geochemical environments across large spatial scales.

### Accession number(s).

This whole-genome shotgun project was deposited in DDBJ/EMBL/GenBank under the accession number NKPT00000000.

## References

[1] Stamps BW, Corsetti FA, Spear JR, Stevenson BS 2014 Draft genome of a novel *Chlorobi* member assembled by tetranucleotide binning of a hot spring metagenome. Genome Announc 2(5):e00897-14. doi:10.1128/genomeA.00897-14. 25212621 PMC4161750

[2] Bryant DA, Costas AMG, Maresca JA, Chew AGM, Klatt CG, Bateson MM, Tallon LJ, Hostetler J, Nelson WC, Heidelberg JF, Ward DM 2007 *Candidatus* Chloracidobacterium thermophilum: an aerobic phototrophic acidobacterium. Science 317:523–526. doi:10.1126/science.1143236. 17656724

[3] Tank M, Bryant DA 2015 *Chloracidobacterium thermophilum* gen. nov., sp. nov.: an anoxygenic microaerophilic chlorophotoheterotrophic acidobacterium. Int J Syst Evol Microbiol 65:1426–1430. doi:10.1099/ijs.0.000113. 25667398

[4] Ward LM 2017 Microbial evolution and the rise of oxygen: the roles of contingency and context in shaping the biosphere through time. Ph.D. dissertation, California Institute of Technology, Pasadena, CA. doi:10.7907/Z9BZ642S.

[5] Li D, Luo R, Liu CM, Leung CM, Ting HF, Sadakane K, Yamashita H, Lam TW 2016 MEGAHIT v1.0: a fast and scalable metagenome assembler driven by advanced methodologies and community practices. Methods 102:3–11. doi:10.1016/j.ymeth.2016.02.020. 27012178

[6] Strous M, Kraft B, Bisdorf R, Tegetmeyer HE 2012 The binning of metagenomic contigs for microbial physiology of mixed cultures. Front Microbiol 3:410. doi:10.3389/fmicb.2012.00410. 23227024 PMC3514610

[7] Aziz RK, Bartels D, Best AA, Dejongh M, Disz T, Edwards RA, Formsma K, Gerdes S, Glass EM, Kubal M, Meyer F, Olsen GJ, Olson R, Osterman AL, Overbeek RA, Mcneil LK, Paarmann D, Paczian T, Parrello B, Pusch GD, Reich C, Stevens R, Vassieva O, Vonstein V, Wilke A, Zagnitko O 2008 The RAST server: rapid annotations using subsystems technology. BMC Genomics 9:75. doi:10.1186/1471-2164-9-75. 18261238 PMC2265698

[8] Parks DH, Imelfort M, Skennerton CT, Hugenholtz P, Tyson GW 2015 CheckM: assessing the quality of microbial genomes recovered from isolates, single cells, and metagenomes. Genome Res 25:1043–1055. doi:10.1101/gr.186072.114. 25977477 PMC4484387

[9] Zeng Y, Feng F, Medová H, Dean J, Koblížek M 2014 Functional type 2 photosynthetic reaction centers found in the rare bacterial phylum *Gemmatimonadetes*. Proc Natl Acad Sci U S A 111:7795–7800. doi:10.1073/pnas.1400295111. 24821787 PMC4040607

